# Improved parallel magnetic resonance imaging reconstruction with multiple variable density sampling

**DOI:** 10.1038/s41598-021-88567-z

**Published:** 2021-04-26

**Authors:** Jinhua Sheng, Yuchen Shi, Qiao Zhang

**Affiliations:** 1 College of Computer Science, Hangzhou Dianzi University, Hangzhou, 310018 Zhejiang China; 2 Key Laboratory of Intelligent Image Analysis for Sensory and Cognitive Health, Ministry of Industry and Information Technology of China, Hangzhou, 310018 Zhejiang China; 3 Beijing Hospital, Beijing, 100730 China

**Keywords:** Biomedical engineering, Electrical and electronic engineering

## Abstract

Generalized auto-calibrating partially parallel acquisitions (GRAPPA) and other parallel Magnetic Resonance Imaging (pMRI) methods restore the unacquired data in k-space by linearly calculating the undersampled data around the missing points. In order to obtain the weight of the linear calculation, a small number of auto-calibration signal (ACS) lines need to be sampled at the center of the k-space. Therefore, the sampling pattern used in this type of method is to full sample data in the middle area and undersample in the outer k-space with nominal reduction factors. In this paper, we propose a novel reconstruction method with a multiple variable density sampling (MVDS) that is different from traditional sampling patterns. Our method can significantly improve the image quality using multiple reduction factors with fewer ACS lines. Specifically, the traditional sampling pattern only uses a single reduction factor to uniformly undersample data in the region outside the ACS, but we use multiple reduction factors. When sampling the k-space data, we keep the ACS lines unchanged, use a smaller reduction factor for undersampling data near the ACS lines and a larger reduction factor for the outermost part of k-space. The error is lower after reconstruction of this region by undersampled data with a smaller reduction factor. The experimental results show that with the same amount of data sampled, using NL-GRAPPA to reconstruct the k-space data sampled by our method can result in lower noise and fewer artifacts than traditional methods. In particular, our method is extremely effective when the number of ACS lines is small.

## Introduction

Parallel Magnetic Resonance Imaging (pMRI) is a technique that uses multiple channels to receive coil information for data acquisition, and "Parallel" means these coils can "sample data at the same time"^1,2^. Compared with traditional MRI, pMRI has improved the speed of data acquisition, so it has been widely used to accelerate medical imaging for the past 20 years. The sampling duration can be reduced by reducing the k-space sampling data as it is determined by the number of phase encoding steps. However, reducing the sampled data will cause severe aliasing after reconstruction, which is a serious problem that must be solved.

Various pMRI techniques have been proposed to accelerate the scan speed without losing image quality. The mainstream methods are roughly divided into two strategies: the imaging algorithm based on image domain and the imaging algorithm based on k-space. The algorithms based on image domain mainly reconstruct the undersampled data into aliased images, and then unfold the aliased images to restore a clear image, such as PILS^3^, SENSE^4^, SC-SENSE^5^, and JSENSE^6^. The algorithms based on k-space are mainly to estimate the missing data in k-space, and then reconstruct the image, such as SMASH^7^, GRAPPA^8^, and improved AUTO-SMASH^9^, VD-AUTO-SMASH^10^, NL-GRAPPA^11^. In recent years, due to the popularity of neural networks, many deep learning approaches have been proposed to accelerate MRI^12,13^. For example, RAKI^14^, SPIRiT-RAKI^15^, rRAKI^16^, DeepSPIRiT^17^ and other methods can reduce artifacts and noise amplification during reconstruction.

GRAPPA is a classical approach for image reconstruction based on k-space domain. GRAPPA assumes that there is a linear relationship between the k-space data, and the coefficient of linear fitting between the k-space data can be estimated from the sampled auto-calibration signal (ACS) lines. The missing k-space data can be fitted by a linear calculation of the required data^8^. The pattern of sampling of GRAPPA is full sampling for the ACS lines in the central k-space while undersampling the other data. The number of ACS lines determines the quality of the reconstruction image. There are model errors and noise-related errors in GRAPPA^18–20^. Thus in recent years, many methods have been proposed to improve the GRAPPA method.

In response to the noise error of GRAPPA, Chang et al. proposed the Nonlinear GRAPPA (NL-GRAPPA). NL-GRAPPA maps the acquired data onto high-dimensional space through a nonlinear transform, and then fit missing data by a linear calculation^11^; Lyu et al. used random projection method to reduce the computing time of GRAPPA^21^. Weller et al. proposed sparsity-promoting regularized calibration method to decrease the effect of noise amplification during reconstruction^22^; Aja‐Fernández et al. proposed to discrimination frequency in ACS lines to discard some points to improve the quality of reconstruction^23^; Lyu proposed a nonlinear framework that uses kernel-based nonlinear methods to improve MRI imaging^24^. Wang et al. proposed the NL-VCC-GRAPPA, which uses virtual conjugate coil (VCC) to reduce noise amplification in GRAPPA^25^. At the same time, someone thought of improving the effect of the reconstruction algorithm by improving the sampling method. Park et al. proposed to use variable density sampling (VDS) to improve the effect of GRAPPA^26^, Zhong et al. proposed to combine GRAPPA and NL-GRAPPA on the basis of VDS to improve the reconstruction effect^27^, Wang et al. proposed Use Crosssampling to improve GRAPPA^28^.

The tradition sampling pattern used by GRAPPA, NL-GRAPPA and other methods, in addition to uniform undersampling data in the outer k-space, some ACS lines are usually sampled at the Nyquist rate in the center of k-space to estimate the linear fitting weights. Traditional sampling pattern use only a single reduction factor for data undersampling in k-space regions outside the ACS region. We proposed a novel reconstruction method with the multiple variable density sampling (MVDS) scheme, which is based on the theory that most of the image information is contained in the central region of k-space^29^. The MVDS method keeps the number of ACS lines in the center of the k-space unchanged while using VDS in the outer k-space for data sampled. In this paper, the method was applied to the NL-GRAPPA, and the results of a set of brain data reconstruction are tested and discussed. In particular, discussion is focused on the effect of undersampling different amounts of data on the reconstruction results by using lower ORF near the ACS lines.

## Results

**Figure 1:**
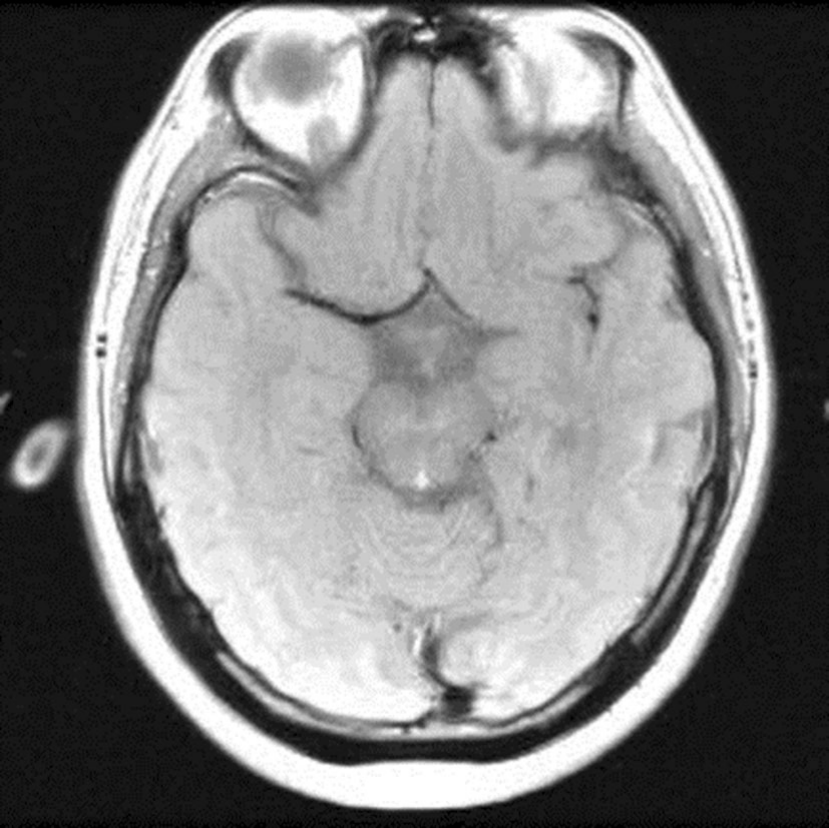
Standard reference brain image reconstructed using SOS.

To demonstrate the effects of the MVDS method proposed in the paper, in vivo data sets were used. Figure 1 shows the standard reference image obtained by using SoS to reconstruct the experimental brain data.

**Figure 2:**
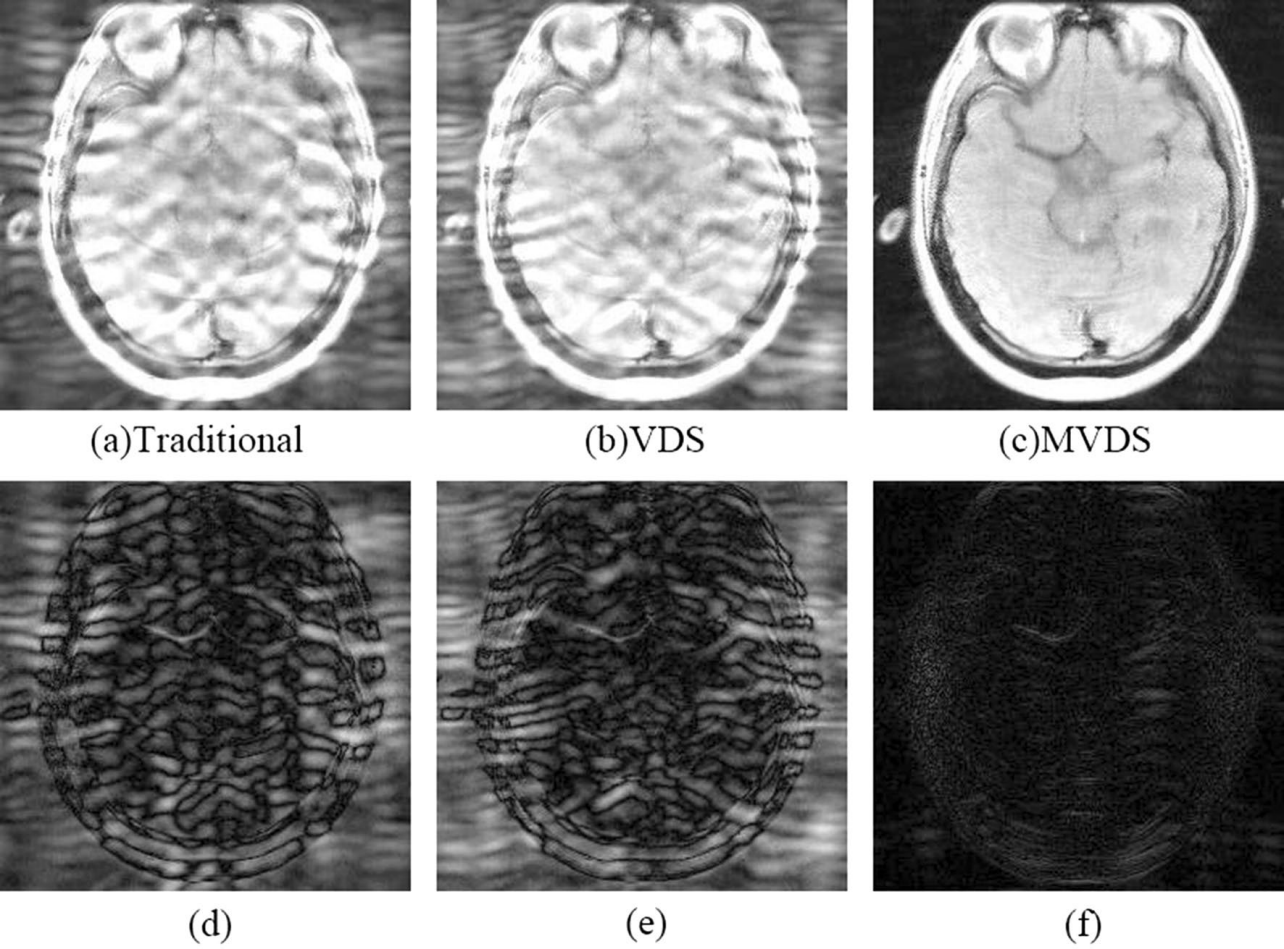
When R = 4 and ACS = 16, use NL-GRAPPA to reconstruct the image to compare traditional, VDS and MVDS methods. (**a**) The traditional method, and (**d**) is the difference graph. (**b**) The VDS method, and (**e**) is the difference map. (**c**) The MVDS method, sampled 20 lines of data with ORF = 2, and (**f**) is the difference graph. Due to the lack of ACS, the performance of the VDS method is worse. The kernel sizes are 15 × 2.

**Figure 3:**
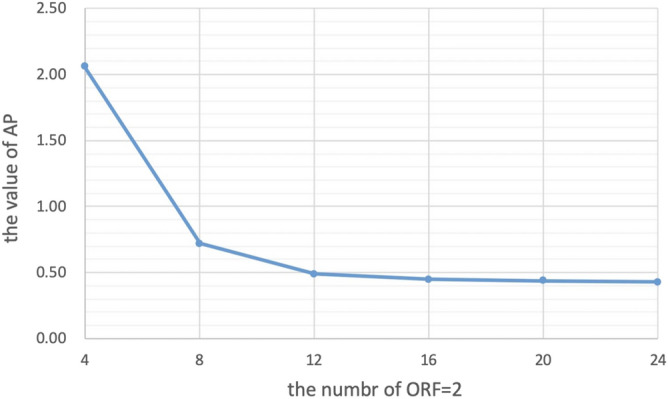
The impact of sampling different numbers of ORF = 2 data on the AP of the reconstruction result image.

Our method is better in the case of lower ACS. When ${\mathrm{R}}_{\mathrm{nom}}$=4 and ACS = 16, the image reconstructed by NL-GRAPPA with traditional sampled method and its different map were shown in Fig. 2(a,d). Used the VDS method to undersample data, reduce the number of ACS lines from 16 to 12, and add 6 lines of under-sampled data with ORF = 2. The kernel sizes are 15 × 2. The reconstructed image and its different map were shown in Fig. 2(b,e). Artifact power (AP) and Signal-To-Noise Ratio (SNR) values were shown in Table 1. We found that too few ACS lines make the reconstruction effect worse than traditional methods. We could see from the image reconstructed by the data sampled by traditional method already have extremely serious artifacts that were completely unrecognizable. Used proposed method to sample data and increase the number of undersampled data with ORF = 2 and 6 from 0. Figure 2(c,f) were the result of undersampling 20 lines of R = 2 and its difference map. The effect of collecting different numbers of lines with ORF = 2 and 6 on the AP value of the reconstructed image was shown in Table 1 and Fig. 3. It can be seen from Fig. 3 that the AP value of the image shows a downward trend as the number of undersampled data with ORF = 2 on both sides of the central area increases. It indicated that the more data collected near the central area with ORF = 2, the better the quality of the reconstructed image. As can be seen from the table, the AP value after reconstruction with the traditional method is 9.59, and with the MVDS method can reduce the AP to 0.44, the effect was obvious. Figure 2(b) was the result of sampling 20 lines with ORF = 2. Compared with the image reconstructed by the traditional method in Fig. 2(a), the artifacts are much reduced. We can see that the MVDS method is obviously better than VDS and traditional methods.

**Table 1:** The comparison of reconstruction images sampled using traditional method, VDS method, and MVDS method.

Methods	Sampled lines				Total lines	${\mathrm{R}}_{\mathrm{net}}$	AP/%	SNR
	ACSL	R = 2	R = 4	R = 6				
Tradition	16	0	60	0	76	3.37	9.59	10.83
VDS	12	6	58	0	76	3.37	12.70	9.76
MVDS	16	4	52	4	76	3.37	2.06	16.93
MVDS	16	8	44	8	76	3.37	0.72	21.42
MVDS	16	12	36	12	76	3.37	0.49	23.11
MVDS	16	16	28	16	76	3.37	0.45	23.42
MVDS	16	20	20	20	76	3.37	0.44	23.53
MVDS	16	24	12	24	76	3.37	0.43	23.60

**Figure 4:**
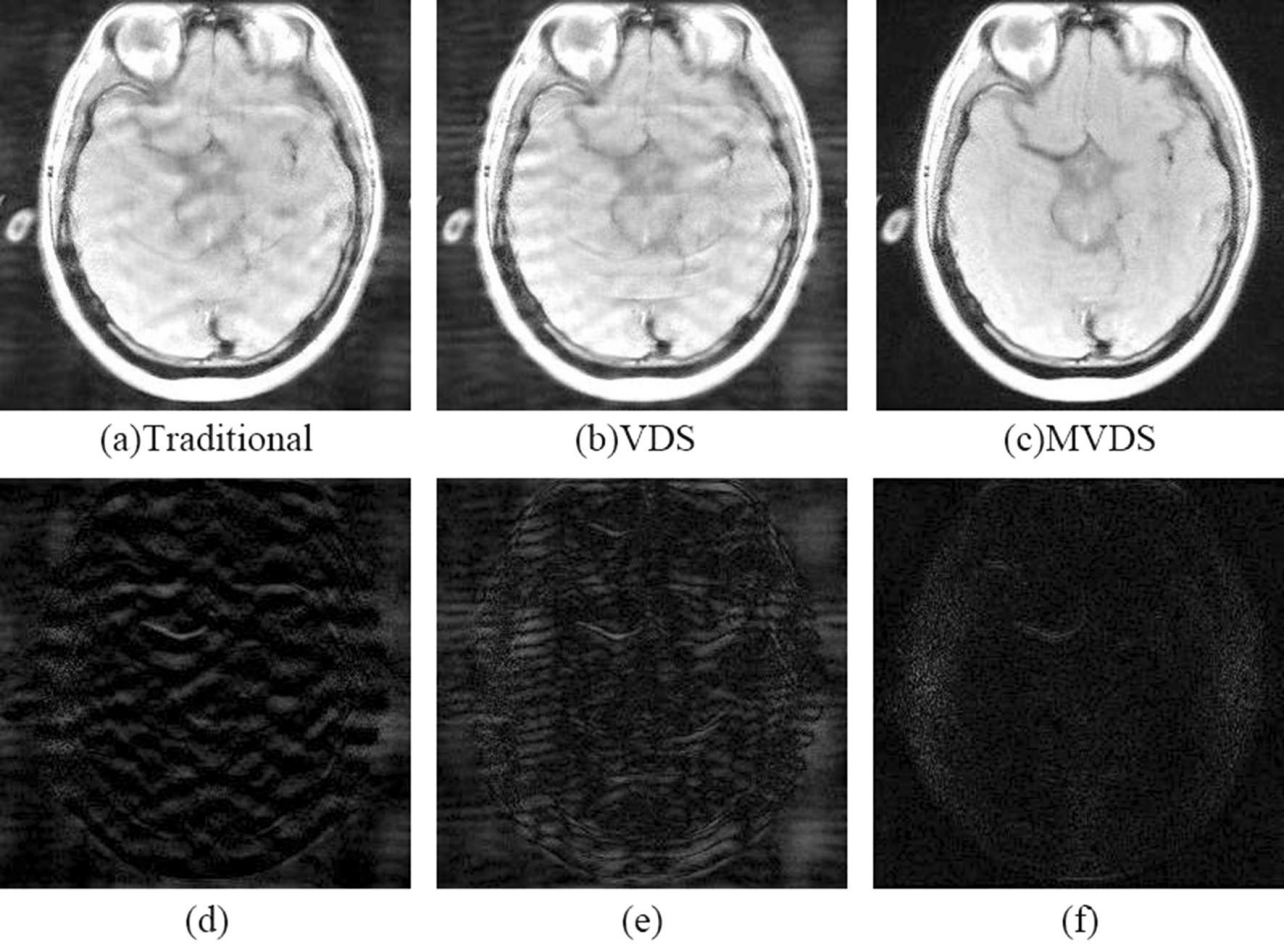
When R = 4 and ACS = 20, use NL-GRAPPA to reconstruct the image to compare traditional, VDS and MVDS method. (**a**) The traditional method, and (**d**) is the difference map. (**b**) The VDS method, and (**e**) is the corresponding difference map. (**c**) The MVDS method, sampling 20 lines of data with ORF = 2, and (**f**) is the difference graph. The kernel sizes are 15 × 2.

**Figure 5:**
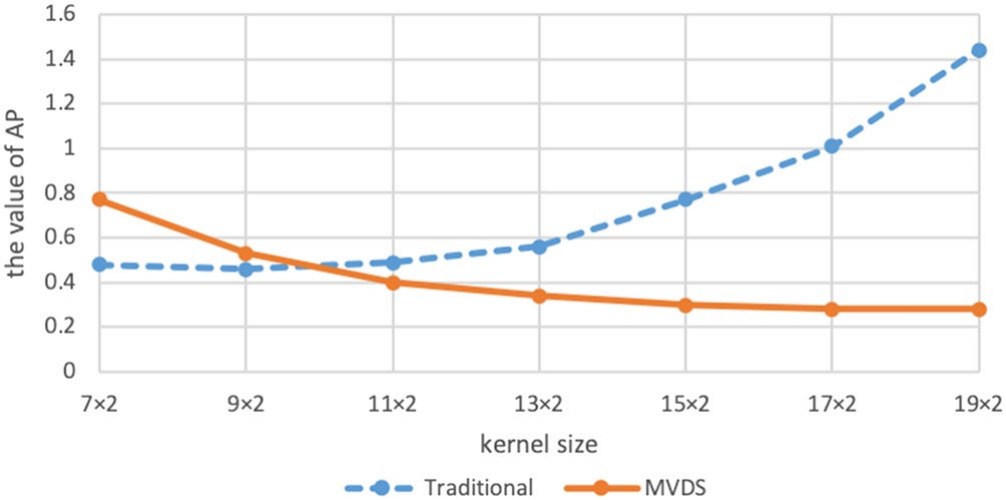
The effect of using different kernel sizes for both MVDS and uniform subsampling.

When ${\mathrm{R}}_{\mathrm{nom}}$=4 and ACS = 20, the image reconstructed by NL-GRAPPA with traditional sampled method and its different map were shown in Fig. 4(a,d). We found that as the ACS decreases, the reconstructed image artifacts become more serious. Using the VDS method to undersample data, reduces the number of ACS lines from 20 to 16, and adds 6 lines of under-sampled data with ORF = 2. The reconstructed image and its different map were shown in Fig. 4(b,e). Its AP and SNR values were shown in Table 2. We found that due to the reduction of ACS lines, the effect is worse than traditional methods. Sampled data with the MVDS method, reduced the ORF on both sides of the ACS data in the central area, use ORF = 2 to undersample data; increase the ORF at the outermost k-space, use ORF = 6 to undersample data. Figure 4(c,f) were the result of undersampling 20 lines of R = 2. From this we can see that the reconstructed image of Fig. 4(c) was clearly superior to the traditional method. Figure 5 showed with the same sampling lines, the AP of reconstruction results using different kernel sizes. We can see that the quality of MVDS method reconstruction is higher under larger kernel size.

**Table 2:** The comparison of reconstruction images sampled using traditional method, VDS method, or MVDS method with different schemes.

Methods	Sampled lines				Total lines	${\mathrm{R}}_{\mathrm{net}}$	AP/%	SNR
	ACSL	R = 2	R = 4	R = 6				
Tradition	20	0	59	0	79	3.24	0.77	21.16
VDS	16	6	57	0	79	3.24	1.12	19.54
MVDS	20	20	19	20	79	3.24	0.30	25.16

**Figure 6:**
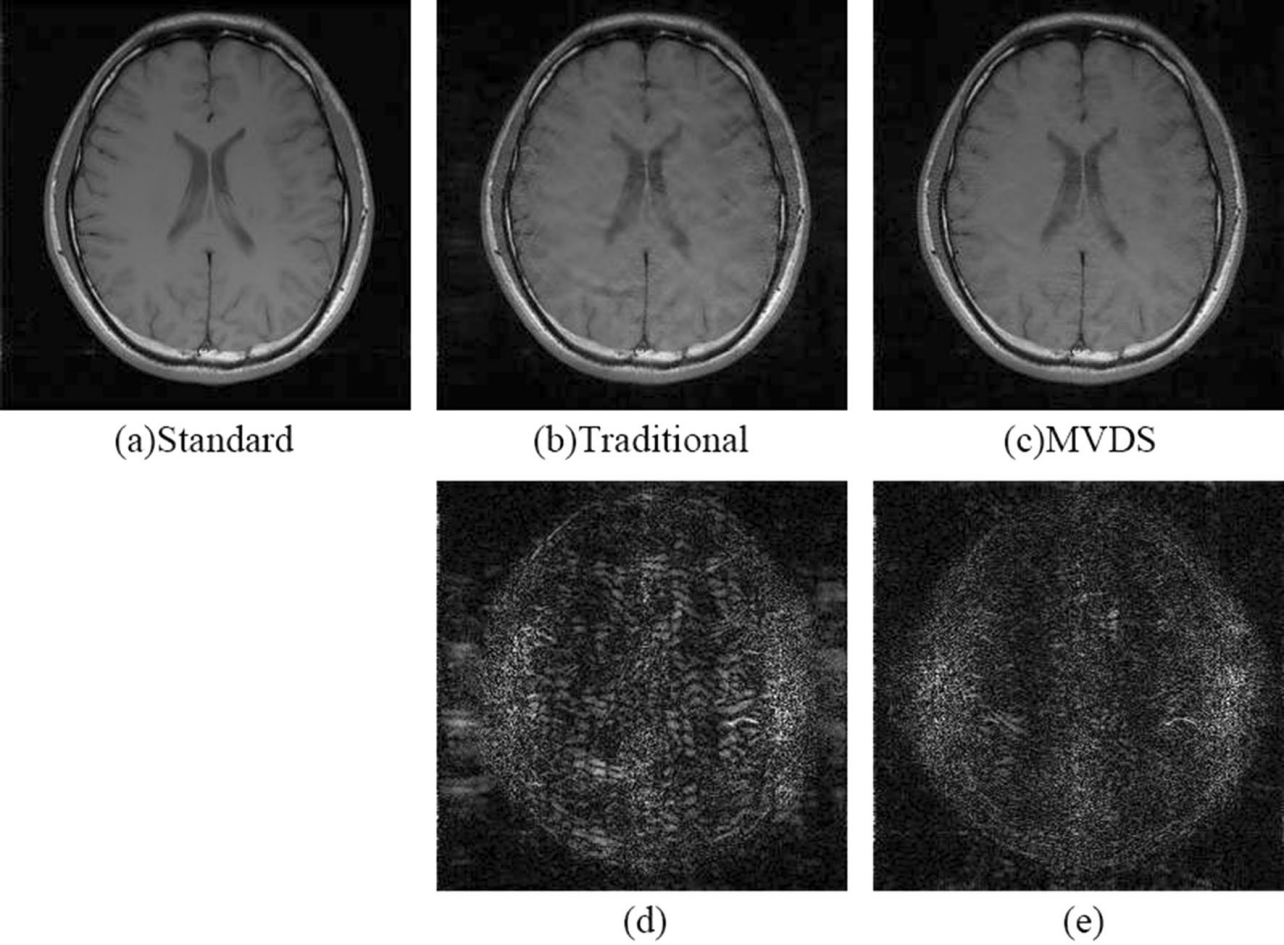
When R = 6 and ACS = 24, use NL-GRAPPA to reconstruct the image to compare traditional and MVDS methods. (**a**) The original standard reference image. (**b**) The traditional method, and (**d**) is the corresponding difference image. (**c**) The MVDS method and sampled 13 lines of data with ORF = 4 and ORF = 8, (**e**) is difference graph.

When ${R}_{nom}$=6 and ACS = 24, another set of eight-channel data^11^ reconstructed by NL-GRAPPA with traditional uniform subsampling and MVDS were shown in Fig. 6. The kernel sizes are 15 × 2. The standard reference image was shown in Fig. 6(a). Figure 6(b,d) are the image reconstructed with traditional sampling method and its different map. Using the proposed MVDS method to subsample data, we increased the ORF from 6 to 8 on the outermost k-space and reduced the ORF from 6 to 4 on the both sides of ACS region. The result image reconstructed with MVDS and its different map were shown in Fig. 6(c,e). The AP and SNR values were shown in Table 3. We could see that the image quality of MVDS method was improved compared with the traditional method.

**Table 3:** The comparison of reconstruction images sampled using traditional methods and MVDS method.

Methods	Sampled lines				Total lines	${\mathrm{R}}_{\mathrm{net}}$	AP/%	SNR
	ACSL	R = 4	R = 6	R = 8				
Tradition	24	0	39	0	63	4.06	0.74	21.33
MVDS	24	13	13	13	63	4.06	0.39	24.13

**Figure 7:**
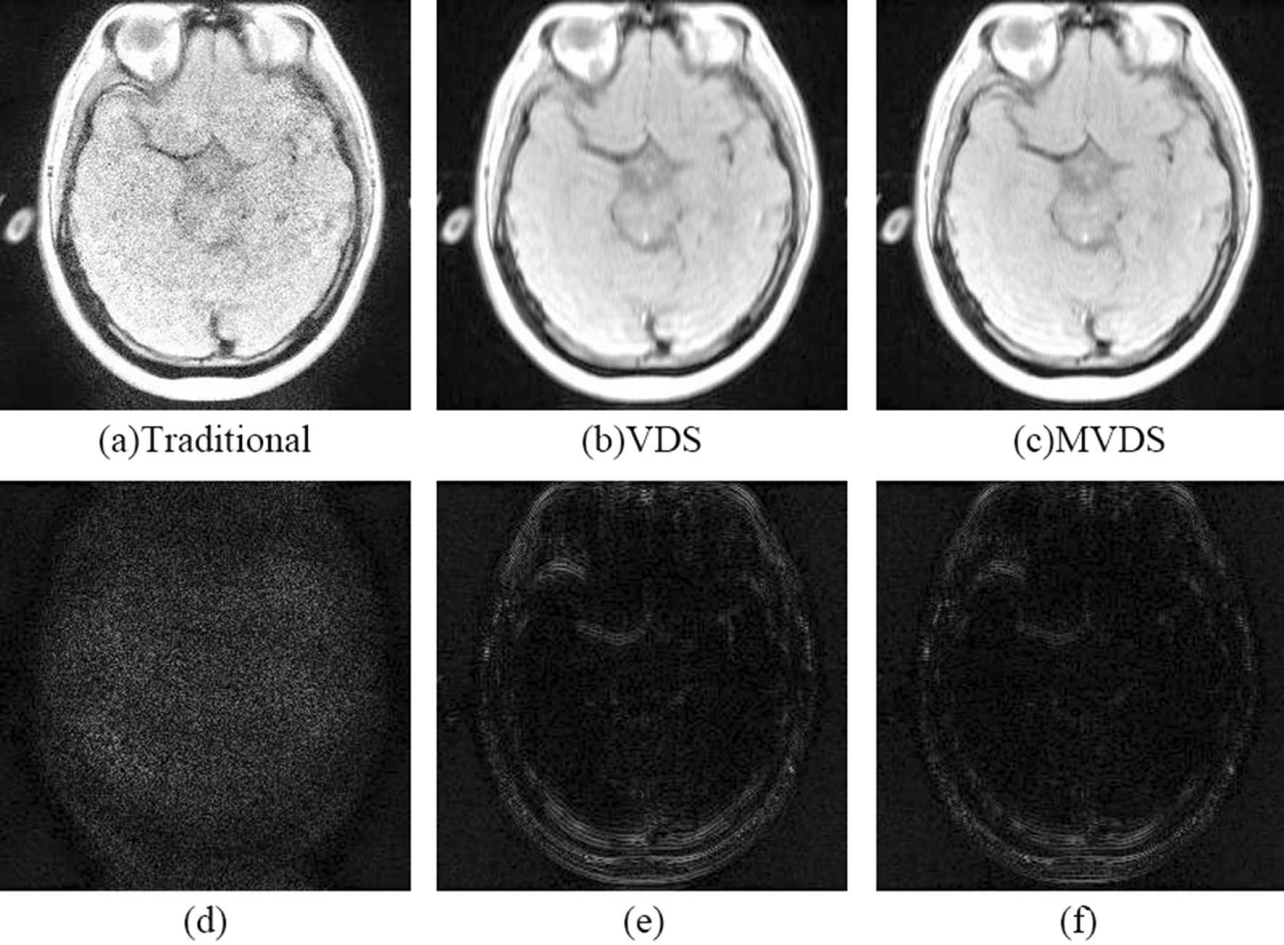
When *R* = 4 and ACS = 40, use GRAPPA to reconstruct the image to compare with traditional, VDS and MVDS methods. (**a**) The traditional method, and (**d**) the difference graph. (**b**) The VDS method, and (**e**) the corresponding difference map. (**c**) The MVDS method and (**f**) the difference graph.

The proposed method was applied to GRAPPA. In addition to NL-GRAPPA, the MVDS method was also effective in GRAPPA. In the case of R = 4 and ACS = 40, the GRAPPA reconstruction images sampled by the three methods were shown in Fig. 7. Figure 7(a,d) were the result image of GRAPPA with traditional sampling method and its difference map. Figure 7(b,c) were the result image with VDS and the proposed method, and Fig. 7(e,f) were their difference maps. The kernel sizes are 15 × 2. The results of sampling number and AP value are shown in Table 4. We still found that the proposed method was better than traditional methods.

**Table 4:** The comparison of GRAPPA reconstruction images sampled using traditional method, VDS method, or MVDS method.

Methods	Sampled lines				Total lines	${\mathrm{R}}_{\mathrm{net}}$	AP/%	SNR
	ACSL	R = 2	R = 4	R = 6				
Tradition	40	0	64	0	94	3.12	1.77	17.65
VDS	32	12	50	0	94	3.12	0.65	21.79
MVDS	40	18	18	18	94	3.12	0.44	23.48

**Figure 8:**
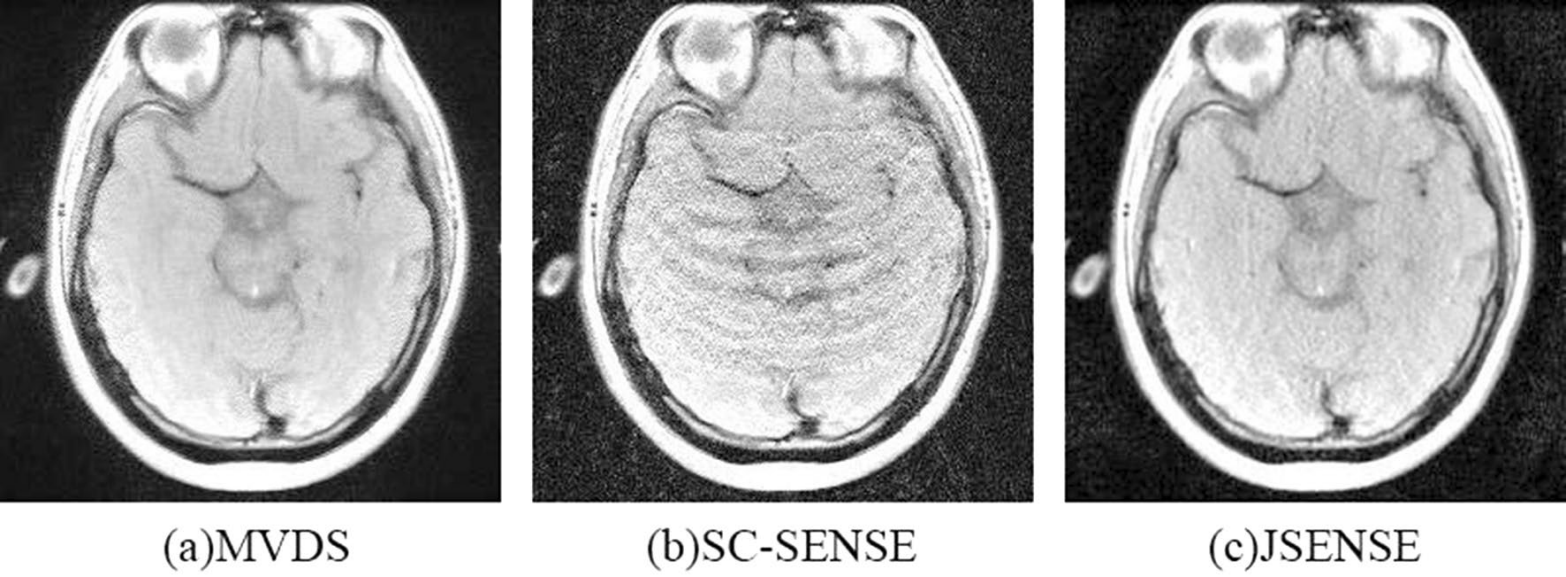
When R = 6 and ACS = 32, compare with other methods. (**a**) Proposed sampling method and the image reconstructed by NL-GRAPPA, kernel size is 15 × 2; (**b**) the SC-SENSE method; (**c**) the JSENSE method.

Compared with other methods, SC-SENSE and JSENSE are well-known image domain reconstruction algorithms. Compared the reconstruction image of proposed method with SC-SENSE and JSENSE, the results were shown in the Fig. 8(a–c). The data in the Table 5 also showed that our image is better than other methods.

**Table 5:** The comparison of reconstruction images using MVDS method, SC-SENSE method and JSENSE method.

Methods	Sampled lines				Total lines	${\mathrm{R}}_{\mathrm{net}}$	AP/%	SNR
	ACSL	R = 2	R = 4	R = 6				
MVDS	32	18	20	18	88	2.91	0.35	24.61
SC-SENSE	32	0	56	0	88	2.91	1.84	17.55
JSENSE	32	0	56	0	88	2.91	0.42	23.75

**Figure 9:**
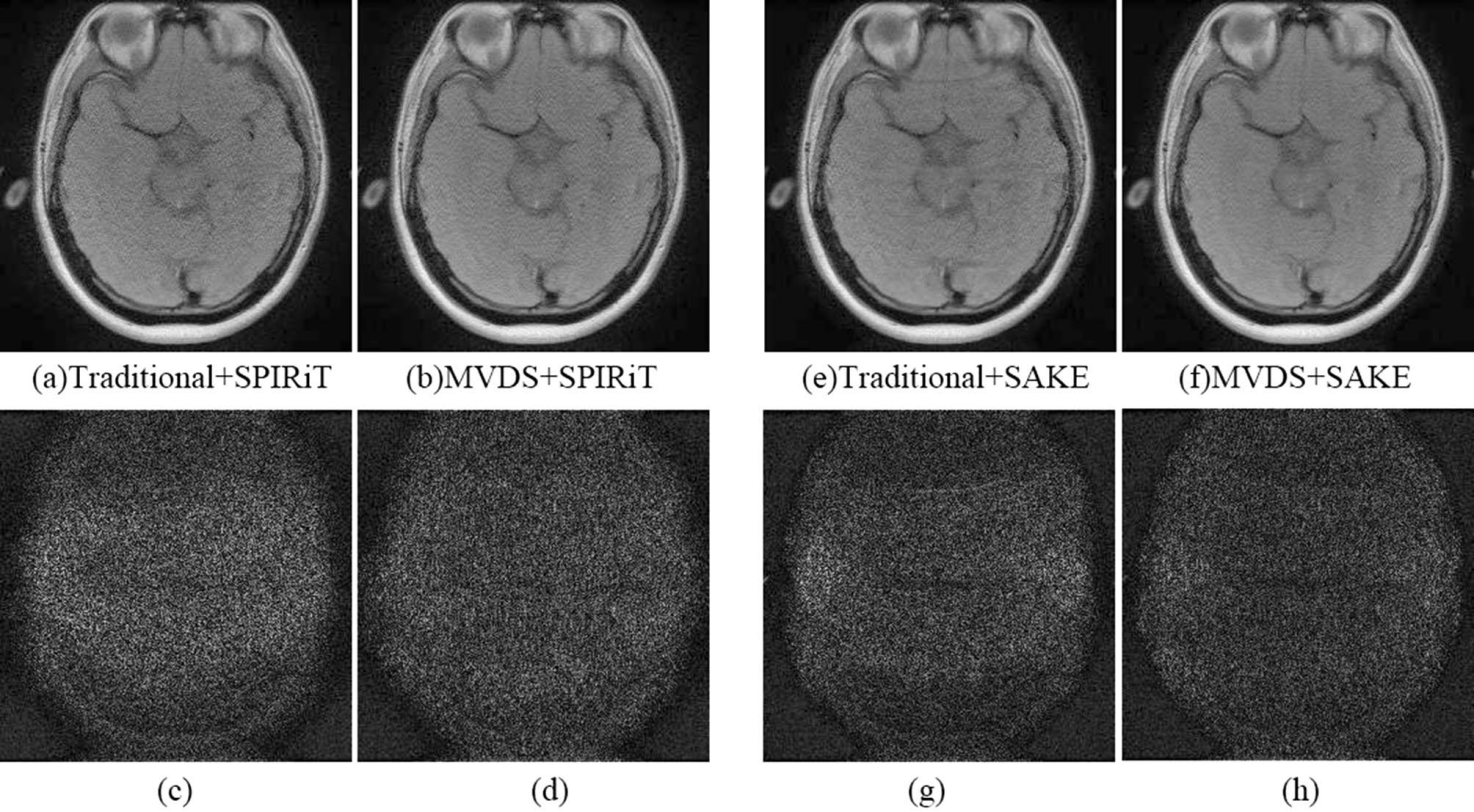
When R = 4 and ACS = 32, use SPIRiT and SAKE to reconstruct the image to compare traditional and MVDS method. SPIRiT: (**a**) the SPIRiT with traditional method and (**c**) the corresponding difference map. (**b**) The SPIRiT with MVDS method, and (**d**) the difference graph; SAKE: (**e**) the SAKE with traditional method and (**g**) the corresponding difference map. (**f**) The SAKE with MVDS method, and (**h**) the difference graph.

SPIRiT is a method that can reconstruct images from arbitrary sampling patterns^30^. SAKE is a calibration data-free parallel imaging reconstruction method^31^. When R = 4 and ACS = 32, the results of SPIRiT and SAKE reconstructed images sampled by traditional uniform subsampling were shown in Fig. 9(a,e), and their different maps were shown in Fig. 9(c,g). The results of SPIRiT and SAKE images sampled by proposed method were shown in Fig. 9(b,f), and their different maps were shown in Fig. 9(d,h). The kernel size in SPIRiT was 5 × 5 and the iterations was 30. The kernel size in SAKE was 6 × 6. We could see that there are still some improvements in the Fig. 9 and Table 6.

**Table 6:** The AP and SNR of the reconstruction images using SPIRiT and SAKE with both traditional uniform subsampling and proposed MVDS.

Methods	Sampled lines				Total lines	${\mathrm{R}}_{\mathrm{net}}$	SPIRiT		SAKE	
	ACSL	R = 2	R = 4	R = 6			AP/%	SNR	AP/%	SNR
Traditional	32	0	56	0	88	2.91	1.14	19.32	0.75	21.17
MVDS	32	18	20	18	88	2.91	0.91	20.52	0.60	22.33

## Discussion

In this paper, we proposed a novel sampling pattern based on an improvement to the traditional sampling pattern. Unlike the traditional sampling pattern, which has only a single reduction factor, we proposed that the sampling pattern includes multiple reduction factors. While keeping the number of ACS unchanged, the MVDS method reduces the value of ORF close to the central k-space by increasing the value of ORF in the outermost k-space, so that the error after reconstruction of the central k-space is smaller. Experimental results prove that the proposed method had resulted in lower noise and fewer artifacts with the same amount of sampled data. At the same time, even when the amount of data sampled by the traditional method is slightly larger, the MVDS method can still maintain the advantages of lower noise and fewer artifacts. The proposed method is more effective at higher reduction factors. At the same time, we only improved the sampling method, and do not involve changes to the reconstruction algorithm. Therefore, it can be directly applied to algorithms similar to the GRAPPA or the algorithms improved from GRAPPA.

The VDS method proposed by Jaeseok increases the number of undersampled data with ORF = 2 by reducing the number of ACS lines in the central area. This method can obviously outperform the traditional method when there are enough ACS lines. But once there are few ACS lines in the traditional method, reducing the number of ACS lines in the VDS method will produce more significant artifacts than the traditional method due to too few ACS lines. Figures 2 and 4(a,b) showed that when there are fewer ACS lines, the image reconstructed by the VDS method is not as good as the traditional method. Figures 2 and 4(a,c) were fully illustrated of the advantages of the MVDS method.

Most of the information of the image is contained in the k-space^29^, so the smaller error in the center of the k-space will enhance image quality. In the method proposed in this paper, the data near the ACS region was sampled with a smaller value of *R*. The reconstruction results with lower ORF have lower noise^11^. The data sampled by these smaller *R* and the ACS lines occupy a wider region of the k-space center as compared to traditional methods that only use ACS lines. Thus, the reconstruction data of this part of the k-space region in the proposed method (lower R) contains less noise than the traditional method (higher R). In the reconstructed images of Figs. 2, 4 and 6 and the results shown in Tables 1, 2 and 3, the advantages of the proposed method were fully demonstrated. More accurate data in central k-space can reduce the noise and artifacts in the reconstruction results.

For the number of sampled lines of different ORFs we can be seen from Fig. 3, after reducing the ORF value close to the ACS area, the AP of the reconstructed image can be reduced, which means that the quality of the image is improved. When the amount of data undersampled with lower ORF in the central area increases, the AP value shows a downward trend. From the Tables 1 and 2, we can see that when the amount of data sampled by different ORFs are similar, the quality of the reconstructed image will be significantly improved compared to the traditional methods. At the same time, it can be seen from Table 1 that when the numbers of lines with three ORFs are similar, changing the number of lines has less of improvement effect on the results. This may be due to the fact that when there are lower ORF data, the number of corresponding higher ORF data must also increase, which makes the area of higher ORF larger and leads to the large error of this part of the area. This part of the increased error has begun to reduce the quality improvement brought by the increase of lower ORF. Although the outermost region contains less information, the larger error of a large number of higher ORF regions will also reduce the quality of the results in the whole image.

Therefore, we recommend that when sampling data, use the lower ORF in the center of k-space to undersample the appropriate data. For example, sampling a similar number of lines from different ORFs may be effective. We can see from Fig. 5 that MVDS will perform better when the kernel is enlarged, and the reconstruction quality is better than the traditional method using the best kernel size.

In order to keep the collection time consistent with traditional methods, we need to increase the ORF in the outermost region. Since the outermost region of k-space contains less information, increasing the ORF value of this region will not have a significant impact on the results. The proposed method does not reduce the number of ACS lines, so it can still maintain its advantages compared with traditional methods when there are fewer ACS. The method proposed in this paper can effectively reduce the artifacts after reconstruction without prolonging the acquisition time (i.e. the same amount of sampling data).

## Material and methods

The source data were drawn from http://bi.tamu.edu/software/downloads_software.htm (University of Texas A&M, USA). To test its performance, in vivo data from a healthy volunteer were acquired on a 3 T MR system (GE Healthcare Technologies, Waukesha, WI, USA) using an eight-channel head coil and a 3D spoiled gradient-echo (SPGR) pulse sequence (TE = 10 ms, TR = 300 ms, RBW = 16 kHz, FOV = 22 cm $\times$ 22 cm, matrix = 256 $\times$ 256)^32^. Informed consent was obtained from the volunteer in accordance with the institutional review board policy. All methods were carried out in accordance with relevant guidelines and regulations. All experimental protocols were approved by the institutional review board (IRB) at Hangzhou Dianzi University (IRB-2020002).

SMASH is an earlier reconstruction approach based on k-space domain. AUTO-SMASH and VD-AUTO-SMASH were proposed to improve this approach. GRAPPA can be seen as a more generalized implementation of VD-AUTO-SMASH^33–35^. The GRAPPA approach needs to acquire a block of extra ACS lines in the central of k-space when undersampling the outer k-space data by some reduction factors (R). The value of the reduction factor R indicates that one line of the data is sampled in every R lines in the outer k-space. These ACS lines are used to determine the complex weights of the linear fit. This estimation process can be simplified as a matrix equation:

1 $${\varvec{b}}={\varvec{A}}{\varvec{x}}$$

where ***A*** represents the matrix of acquired data of ACS line, ***b*** represents the "missing data" but are actually acquired from ACS lines, and ***x*** represents the linear weights to be fitted.

**Figure 10:**
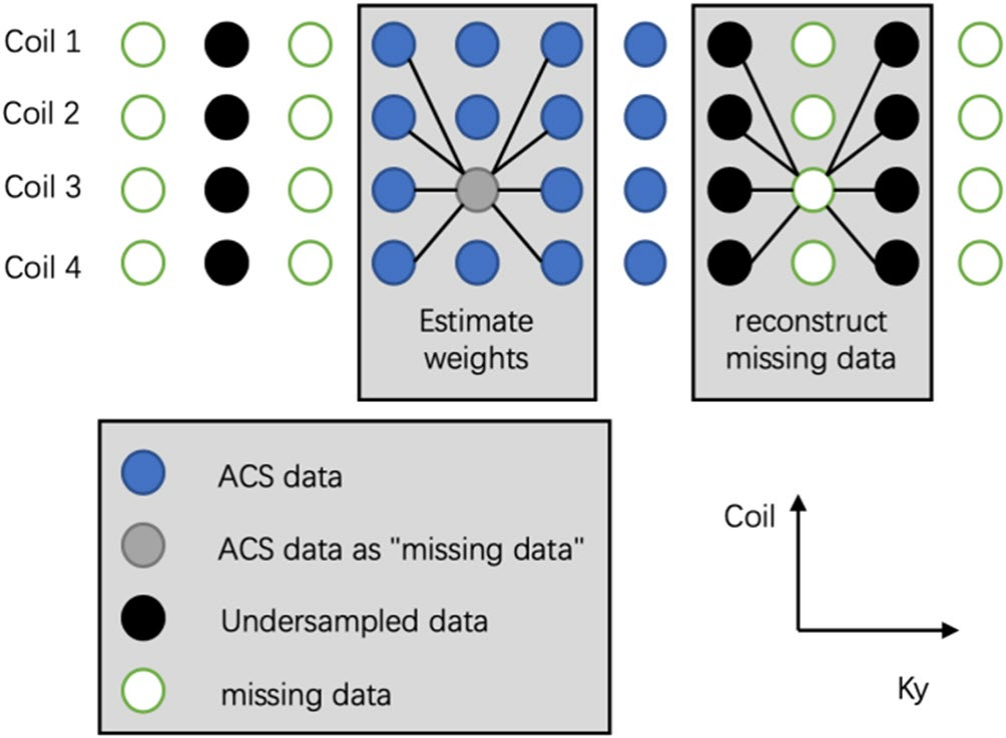
The basic principle of the GRAPPA algorithm is shown above. The sampled data around a sampled point in the ACS data is used to calculate the weight of these sampled points to the point. Then use the sampled data around the missing point to use the weight to calculate the value of the missing point. ORF = 2 in this case.

The estimation process was shown in the Fig. 10, we used the ACS data around the "missing data" to fit the "missing data" to estimate the weights, and then used theses weights to estimate the truly missing data from the undersampled data.

NL-GRAPPA improves on traditional GRAPPA and introduces extra nonlinear mapping steps. Specifically, the undersampled k-space data *A* are mapped to a high-dimensional space *H* through a nonlinear mapping Φ (·): *A*- > *H*, and then the unacquired data is reconstructed. The weights of the linear fit can also be estimated from the ACS data. The relationship between the selected kernel and the mapping *Φ* is as follows:

2 $$k\left({a}_{1},{a}_{2}\right)=<\Phi \left({a}_{1}\right),\Phi \left({a}_{2}\right)>, \forall {a}_{1},{a}_{2}\in A$$

where <,> represents the inner product. There are many different types of kernels^36^. This method chooses the a polynomial kernel^11,37^, it can be expressed as

3 $$k\left({a}_{i},{a}_{j}\right)={\left(\gamma {a}_{i}^{T}{a}_{j}+r\right)}^{d}$$

where the parameter value is γ = *r* = 1 and *d* = 2.

The formula of nonlinear GRAPPA method using this kernel can be expressed as follows:

4 $$ \begin{aligned}   S_{j} \left( {k_{y}  + r\Delta k_{y} ,k_{x} } \right) &  = w_{{j,r}}^{{\left( 0 \right)}}  \times 1 + \sum\limits_{{l = 1}}^{L} {\sum\limits_{{b = B_{1} }}^{{B_{2} }} {\sum\limits_{{h = H_{1} }}^{{H_{2} }} {w_{{j,r}}^{{\left( 1 \right)}} } } } \left( {l,b,h} \right) \times S_{l} \left( {k_{y}  + bR\Delta k_{y} ,k_{x}  + h\Delta k_{x} } \right) \\     &  \quad+ \sum\limits_{{l = 1}}^{L} {\sum\limits_{{b = B_{1} }}^{{B_{2} }} {\sum\limits_{{h = H_{1} }}^{{H_{2} }} {w_{{j,r}}^{{\left( {{\text{2}},{\text{0}}} \right)}} } } } \left( {l,b,h} \right) \times S_{l}^{2} \left( {k_{y}  + bR\Delta k_{y} ,k_{x}  + h\Delta k_{x} } \right) \\     &  \quad+ \sum\limits_{{l = 1}}^{L} {\sum\limits_{{b = B_{1} }}^{{B_{2} }} {\sum\limits_{{h = H_{1} }}^{{H_{2} }} {w_{{j,r}}^{{\left( {{\text{2}},{\text{1}}} \right)}} } } } \left( {l,b,h} \right) \times S_{l} \left( {k_{y}  + bR\Delta k_{y} ,k_{x}  + h\Delta k_{x} } \right) \times S_{l} \left( {k_{y}  + bR\Delta k_{y} ,k_{x}  + \left( {h + 1} \right)\Delta k_{x} } \right) \\     &  \quad+ \sum\limits_{{l = 1}}^{L} {\sum\limits_{{b = B_{1} }}^{{B_{2} }} {\sum\limits_{{h = H_{1} }}^{{H_{2} }} {w_{{j,r}}^{{\left( {{\text{2}},{\text{2}}} \right)}} } } } \left( {l,b,h} \right) \times S_{l} \left( {k_{y}  + bR\Delta k_{y} ,k_{x}  + h\Delta k_{x} } \right) \times S_{l} \left( {k_{y}  + bR\Delta k_{y} ,k_{x}  + \left( {h + 2} \right)\Delta k_{x} } \right) \\  \end{aligned}  $$

where *S*_*j*_(*k*_*y*_ + *r*∆*k*_*y*_,*k*_*x*_) denotes the missing k-space data at the target coil, *S*_*j*_(*k*_*y*_ + *tR*∆*k*_*y*_,h∆*k*_*x*_) denotes the acquired undersampled data, and w_*i*,*j*_(*l*,*t*,*h*)denotes the weights of linear combination. The first-order term is equivalent to the conventional GRAPPA, and the second-order term can represent other nonlinear models. Here *R* represents the reduction factors, *l* counts all coils, *t* and *h* represents the number of reconstruction blocks, k_x_ and k_y_ represent the coordinates along the frequency-encoding and phase-encoding directions^11^.

At the same time, the formula for estimating the fitting weights *b* = *Ax* can be transformed to

$$b=\Phi \left(A\right)x$$

5 $$\Phi \left(A\right)={\left[\Phi \left({{\varvec{a}}}_{1}\right),\Phi \left({{\varvec{a}}}_{2}\right),\cdots ,\Phi \left({{\varvec{a}}}_{{\varvec{M}}}\right)\right]}^{T}$$

where the matrix Φ(*A*) is size of M × N_k_, M represents the number of all matching equations during estimation, N_k_ is the dimension of the new high-dimensional feature space, and a_i_ is the row vector of *A*.

### Traditional sample pattern

**Figure 11:**
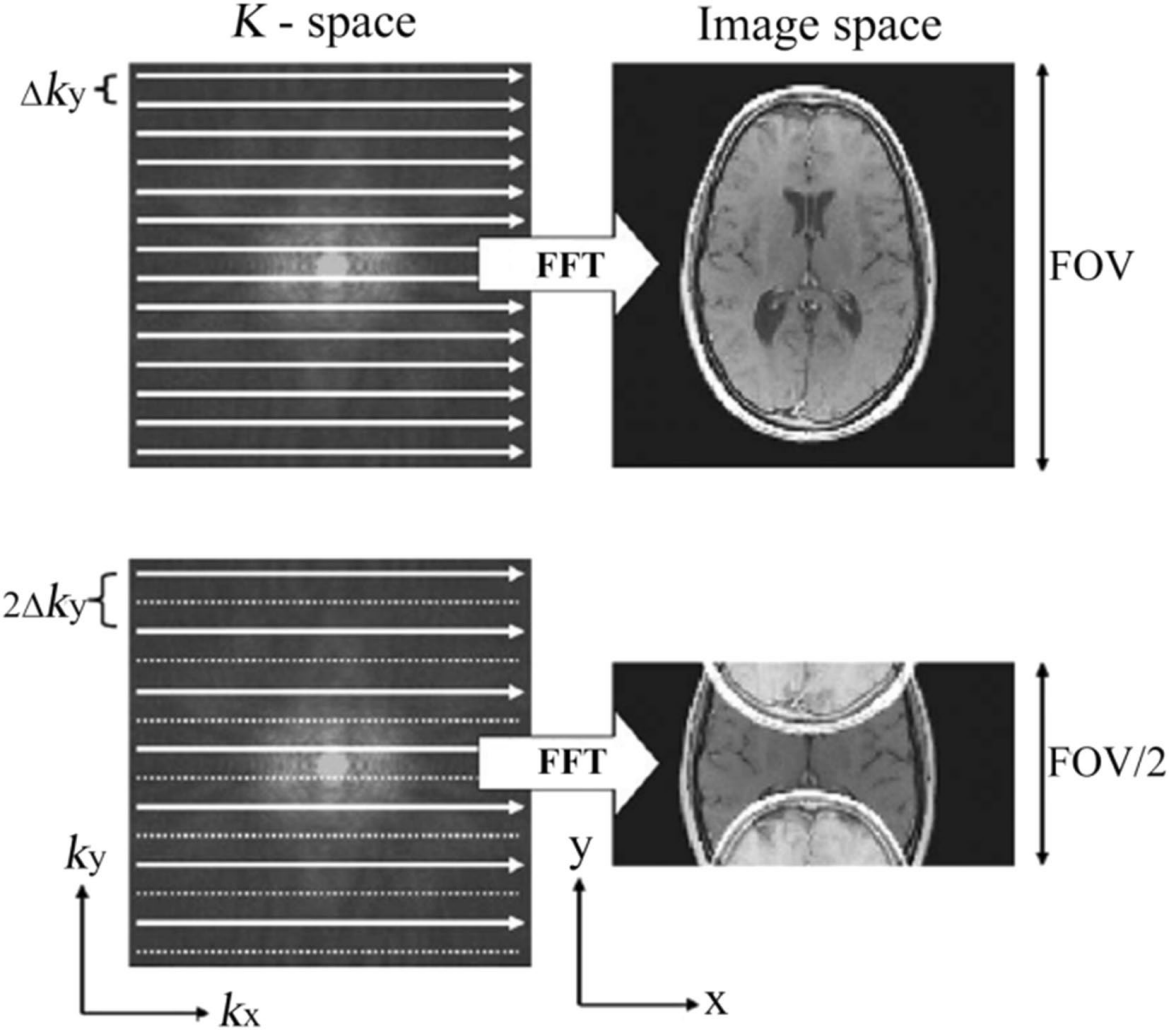
Comparison of fully sampled acquisition (top row) and undersampled acquisition with the acceleration factor of 2 (bottom row). Dot lines mean missing lines; solid lines are acquired lines. FFT means Fast Fourier Transformation.

The data to fill the k-space is taken directly from the MR signal. But sampling in the full space is very slow. Because frequency encoding steps are much faster than phase encoding steps in MRI scanners, one way to accelerate MRI data acquisition is to reduce the number of phase encoding lines. This type of undersampled dataset is equal to fold images along phase encoding direction and yield aliasing artifacts along the undersampled direction with a reduced field of view (FOV) as seen as Fig. 11. Fully sampling results in a full FOV image after Fourier transformation, and undersampling yields a reduced FOV image with aliasing artifacts.

The traditional sampling pattern is to choose a certain reduction factor for uniform undersampling to acquire data. The sampling pattern of SC-SENSE, GRAPPA, NL-GRAPPA and other methods are similar. While sampling the undersampled data, some ACS data need to be obtained at the Nyquist rate in the central k-space. SC-SENSE uses these ACS data to extract a more accurate sensitivity calibration images. GRAPPA, NL-GRAPPA uses these ACS lines to calculate the coefficients of the linear fit. We use the expected reduction factor (termed nominal reduction factor, ${\mathrm{R}}_{\mathrm{nom}}$) to represent the outer reduction factor (ORF) used in traditional methods, which means that data is collected once every ORF rows.

### Variable density sampling

**Figure 12:**
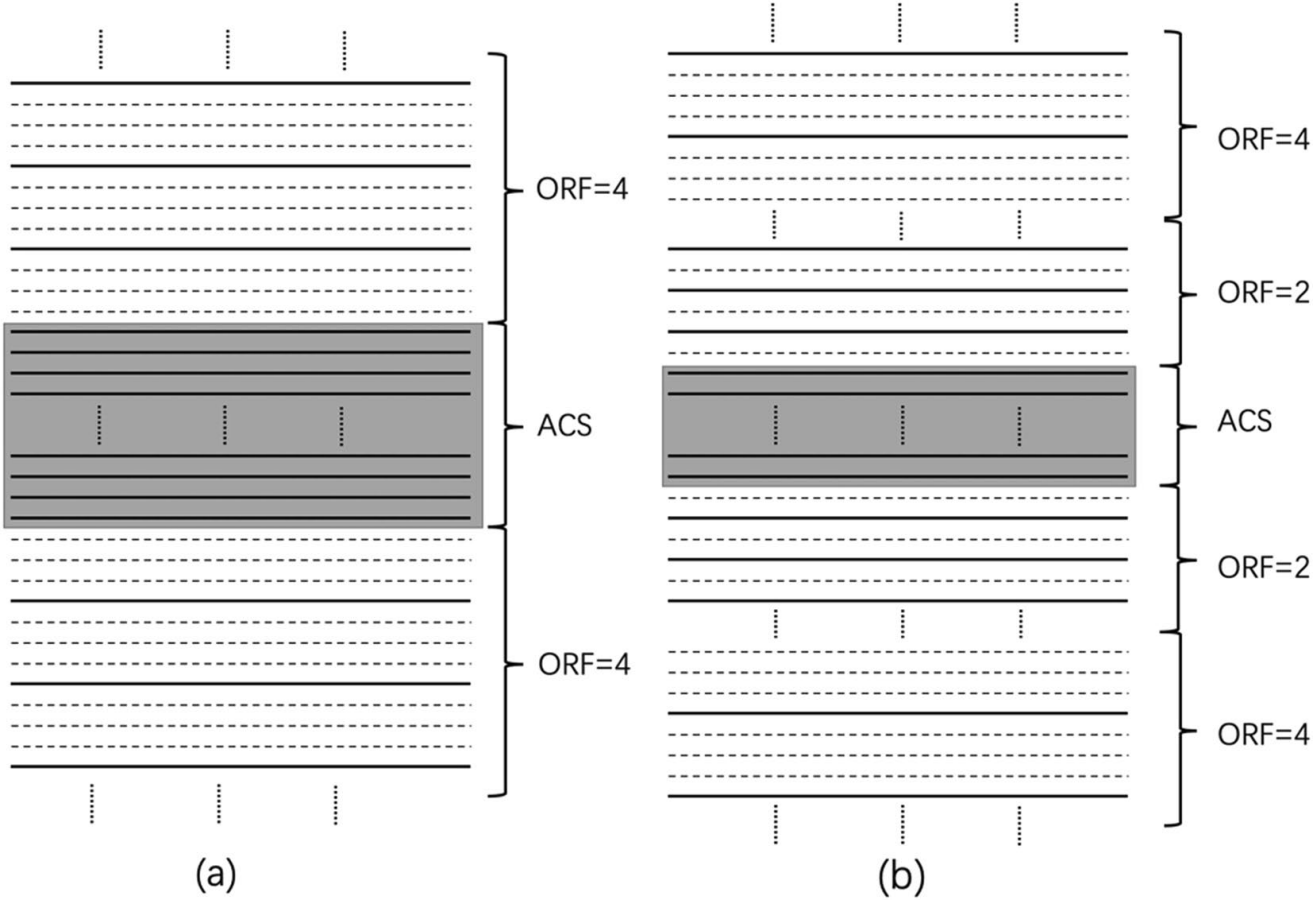
The picture shows the case of ORF = 4. (**a**) The traditional method, the central area is fully sampled, and the outer k-space uses ORF = 4 for uniform undersampling; (**b**) the VDS method. The total sampling line in the central area is less than that of (**a**), but ORF = 2 is added on both sides for undersampling, and the outermost area uses ORF = 4 for uniform undersampling.

Park et al. improved the traditional sampling method^26^, using a lower ORF on both sides of the ACS lines for undersampling data. As shown in the Fig. 12(a) is the traditional sampling method and (b) is the method proposed by Park et al., and the ${\mathrm{R}}_{\mathrm{nom}}$=4. In Fig. 12(b), Park et al. increased the undersampled data with ORF = 2 on both sides of the central area by reducing the number of full sampled ACS lines. Note that the full sampled ACS lines as well as the data undersampled with ORF = 2 is larger than the k-space occupied by the ACS lines in (a). And this part of the area occupies most of the central area of k-space. Therefore, the error after reconstruction is small, and the image quality will be improved.

The VDS method proposed by Park et al. keeps the sampling time consistent with the traditional method by reducing the number of ACS lines sampled. The redundant time of sampling ACS data will be reduced to sample the data with ORF = 2. In this way, the error of reconstructed data at the ORF = 2 area becomes smaller. It makes the reconstruction image quality better than the traditional method. However, once the number of ACS lines in the traditional method is very small (for example, ACS = 24, ACS = 16), then reducing the ACS lines will make the results produce extremely significant artifacts. Therefore, we propose a method to keep the number of sampled ACS lines consistent with the traditional method and increase the value of ORF in outermost k-space to reduce the ORF value on both sides of the ACS area. The improved method is applied to NL-GRAPPA, and the effect is much better than the traditional method in the case of lower ACS lines.

### Multiple variable density sampling

**Figure 13:**
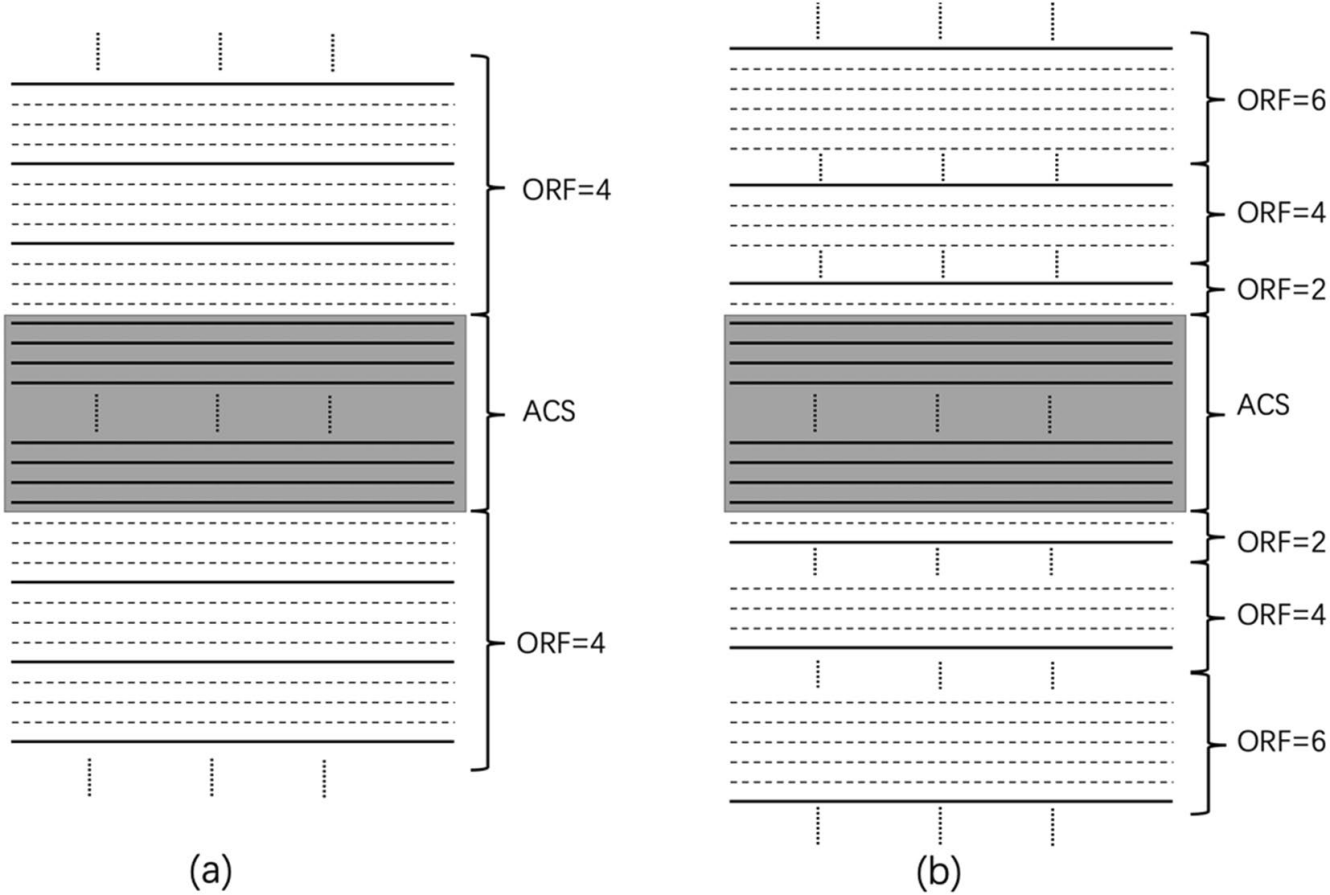
The picture shows the case of ORF = 4. (**a**) The traditional method, the central area is fully sampled, and the outer area uses ORF = 4 for uniform undersampling; (**b**) MVDS method, the central area is fully sampled, the ORF on both sides of the central area is reduced from 4 to 2, and the outermost ORF is increased from 4 to 6 in order to keep the sampling time unchanged. The number of ACS data collected in the central area of the MVDS method is consistent with the traditional method.

We proposed a novel reconstruction method with the MVDS scheme. The MVDS method keeps the number of ACS lines in the center of the k-space unchanged while using VDS in the outer k-space for data sampled. The proposed method was based on the theory that higher reduction factors will cause higher errors and that most of the image information is contained in the central area of k-space. The MVDS method specifically increases the value of the ORF of the data undersampled in the outermost k-space to decrease the value of the ORF undersampled near the ACS lines. The number of ACS lines and the total amount of data sampled remain unchanged. The data undersampled using this method will be more accurate near the center of k-space after reconstruction due to the lower ORF. This method was shown in Fig. 13. Taking ${\mathrm{R}}_{\mathrm{nom}}$=4 as an example, Fig. 13(a) was the traditional sampling method, and Fig. 13(b) showed the MVDS method proposed in this paper. Please note that the number of ACS lines fully sampled by this method is consistent with the traditional method. In the traditional method, we increase the ORF = 4 to R = 6 in the outermost k-space, and reduce the ORF = 4 to ORF = 2 on both sides of the ACS region. In k-space, most of the image information is in the central region. Therefore, a lower ORF value in the central region will make the image error smaller. Unlike the VDS method in Fig. 12(b), the method was to keep the outermost R = 4 unchanged, and reduce the number of ACS lines sampled at the innermost side to reduce the value of ORF on both sides of the lower central area from 4 to 2 to keep the sampling time unchanged. But we reduced the value of ORF on both sides of the central area while we increased the value of ORF in the outermost k-space to keep the sampling time unchanged. The MVDS method does not change the amount of ACS lines, so it can also produce excellent results when the traditional method collects fewer ACS lines.

The difference between the MVDS method and the traditional method is that we do not change the number of ACS lines. When using ACS data to calculate weights, more ACS lines make the calculated weights more accurate, and too few ACS lines will produce significant artifacts. Therefore, when the ACS line is sufficient in the VDS method, the reconstruction data error of ORF = 2 area is smaller and the reconstruction result is better. But once the ACS lines used in the traditional method are less, reducing the number of ACS data will produce more serious artifacts to the result. The MVDS method does not change the number of ACS lines, and reduces the ORF value in the central area. This part of the area will have a lower error than the traditional method after restoration. Therefore, the MVDS method can produce excellent results when fewer ACS lines are collected.

**Figure 14:**
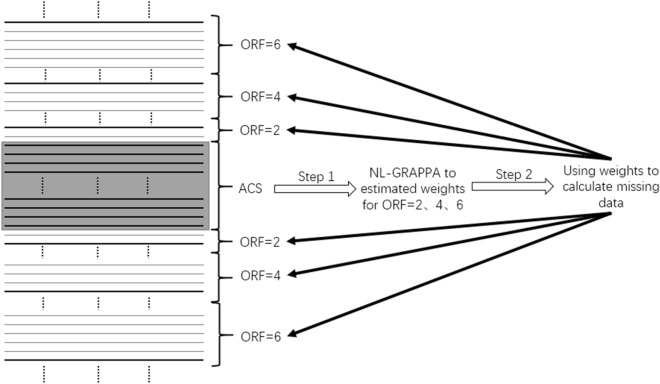
The process of restoring the complete data of k-space. The thick solid line is the sampling data, and the thin solid line is the missing data restored.

The method of restoring the missing k-space data using the data sampled by the MVDS method is similar to the traditional method. As shown in Fig. 14, the first step was to use the corresponding reconstruction method (GRAPPA or NL-GRAPPA, etc.) to estimate the weight of the corresponding ORF, and the second step was to use the weight to calculate the missing data of the corresponding region. In Fig. 5, the thick line was the sampling data, and the thin line is the missing value restored by the reconstruction method. After these two steps, the complete k-space data can be obtained.

The proposed method in this paper was based on the theory that most image information, including contrast and general shape, was contained in the center of space^29^. We know that a higher acceleration factor will lead to higher noise^11^. Therefore, if undersampling the data in the region near the center with a lower reduction factor, the error in this region will be smaller than traditional after reconstruction. In the traditional method, if the reduction factor is high, the region of reconstruction data except ACS lines will have a large error. The proposed method kept the ACS lines unchanged and sampled data with lower ORF values on both sides of the central area. Therefore, the error in the center of k-space is smaller and the reconstructed image is more accurate. A higher ORF is used for sampling the outermost area of the k-space region to keep the total acquisition time consistent, but it has little influence on the reconstruction images because of the less information contained in this part of the region. The MVDS method is superior to the traditional method when the number of ACS lines is fewer.

### Image acquisition and analysis

The full sample k-space data of the eight coils were first to generate the corresponding coil image by performing inverse Fourier transform. We used the sum of squares method to process the images of eight coils and generated the SoS image as a standard image reference. The calculation method is as follows:

6 $${I}_{sos}=\sqrt{\sum_{i=1}^{CoilNum}{I}_{j}{I}_{j}^{T}}$$

In the formula, ${I}_{j}$ represents an image generated by inverse Fourier transform of k-space data of each coil, CoilNum represents the number of coils, and ${I}_{sos}$ represents the generated standard image reference.

Each pixel in each reconstruction image is subtracted from the corresponding pixels in the SoS image to obtain equal scale difference maps. These maps can more directly show the severity and distribution of noise and the location of artifacts. In this experiment, the AP and SNR were used to measure the quality of the reconstructed image. The AP is used to describe the error between the reconstructed image and the standard reference image. A higher AP value indicates the reconstructed image has greater noise. The calculation method of AP is as follows:

7 $$AP=\frac{\sum_{\left(x,y\right)\in ROI}{||{I}^{reference}\left(x,y\right)|-|{I}^{recon}\left(x,y\right)||}^{2}}{\sum_{\left(x,y\right)\in ROI}{\left|{I}^{reference}(x,y)\right|}^{2}}.$$

where the I^recon^ represents the reconstructed image, I^ref^ represents the standard reference image, and ROI means the region of interest. In this experiment, we selected the entire image matrix region, which is ROI = 256. It can be seen from the formula that a smaller the value of AP means the reconstructed image is closer to the standard reference image, that is, the higher the reconstruction error.

The signal-to-noise ratio SNR of an image is also an indicator to measure the quality of the image. The larger the SNR value indicates the less noise of the reconstructed image. The formula is as follows:

8 $$SNR=10*{\mathrm{log}}_{10}\left(\frac{\sum_{\left(x,y\right)\in ROI}{\left|{I}^{recon}\left(x,y\right)\right|}^{2}}{\sum_{\left(x,y\right)\in ROI}{\left|{I}^{recon}\left(x,y\right)-{I}^{reference}\left(x,y\right)\right|}^{2}}\right)$$

In pMRI we use the value of reduction factor to indicate the degree of pMRI being accelerated. The termed nominal reduction factor (${\mathrm{R}}_{\mathrm{nom}}$) represents that sample k-space data every R lines. The larger the value of ${\mathrm{R}}_{\mathrm{nom}}$ means the less the total amount of data sampled, and the less time pMRI costs. Some methods such as GRAPPA need to sample extra ACS data in the central k-space, leading to an increase in the total amount of data sampled. These increase data make the actual net reduction factor (${\mathrm{R}}_{\mathrm{net}}$) and ${\mathrm{R}}_{\mathrm{nom}}$ different. In the traditional sampling method, the ACS lines are fully sampled in the center of k-space (R = 1), and the ${\mathrm{R}}_{\mathrm{nom}}$ is used to sample data in the outer k-space region. Therefore, ${\mathrm{R}}_{\mathrm{net}}$ is usually smaller than ${\mathrm{R}}_{\mathrm{nom}}$ that we expected. The number of sampled lines and the calculation method of ${\mathrm{R}}_{\mathrm{net}}$ are as follows:

$$\mathrm{SUM}={N}^{ACSL}+\frac{{N}^{fully\,sampled}-{N}^{ACSL}}{{R}_{nom}}$$

9 $${R}_{net}=\frac{{N}^{fully\,sampled}}{SUM}$$

where N^fully^ represents the total number of lines of k-space data, and N^ACSL^ represents the number of extra ACS lines. SUM represents the amount of data lines sampled.

## Conclusion

We proposed an MVDS method that uses multiple reduction factors in the sampling process to improve the reconstruction quality of pMRI. Areas sampled with a factor lower than the nominal reduction factor can have less noise. The proposed method effectively reduces noise and artifacts, especially when there are fewer ACS lines. We apply the proposed method to NL-GRAPPA and GRAPPA, and compare them with traditional sampling methods, SC-SENSE, JSENSE and other methods. The experimental results show that this method has obvious advantages compared with the traditional sampling method and VDS method. This method only improves the sampling pattern, and does not change the algorithm. The sampling pattern introduced here can be used in most existing technologies similar to GRAPPA. At the same time, our method has some shortcomings. For example, it is currently only suitable for k-space based imaging algorithms. Our method needs to be improved overtime with the goal of achieving a more universal method.
